# Serum and Erythrocyte Antioxidant Defense in Colorectal Cancer Patients during Early Postoperative Period: Potential Modifiers and Impact on Clinical Outcomes

**DOI:** 10.3390/antiox10070999

**Published:** 2021-06-23

**Authors:** Małgorzata Krzystek-Korpacka, Magdalena Mierzchała-Pasierb, Marek Zawadzki, Dorota Diakowska, Wojciech Witkiewicz

**Affiliations:** 1 Department of Biochemistry and Immunochemistry, Wroclaw Medical University, 50-368 Wroclaw, Poland; magdalena.mierzchala-pasierb@umed.wroc.pl; 2 Department of Oncological Surgery, Regional Specialist Hospital, 51-124 Wroclaw, Poland; zawadzki@wssk.wroc.pl (M.Z.); witkiewicz@wssk.wroc.pl (W.W.); 3 Department of Physiotherapy, Wroclaw Medical University, 51-618 Wroclaw, Poland; 4 Department of Nervous System Diseases, Wroclaw Medical University, 51-618 Wroclaw, Poland; dorota.diakowska@umed.wroc.pl; 5 Research and Development Centre at Regional Specialist Hospital, 51-124 Wroclaw, Poland

**Keywords:** colorectal cancer, ferric reducing antioxidant power (FRAP), total antioxidant capacity, superoxide dismutase, glutathione peroxidase, surgical trauma, robotic surgery, postoperative ileus, anastomotic leak, antioxidant intervention

## Abstract

A better understanding of antioxidant status, its modifiers, and its effect on clinical outcomes in patients undergoing colorectal cancer surgery is needed for effective antioxidant-based interventions. The objectives of this cohort study were: to determine baseline serum (total antioxidant capacity (TAC) and ferric reducing antioxidant power (FRAP); *n* = 72) and erythrocyte (superoxide dismutase (SOD) and glutathione peroxidase (GPx); *n* = 47) antioxidant capacity and time-course during the 72 h postoperative period, to identify potential modifiers, and to establish impact on clinical outcomes. Older patients with comorbidities had lower baseline FRAP. TAC was inversely and SOD directly correlated with inflammatory markers. Cancer pathology affected GPx (lower in advanced and more aggressive cancers) and SOD (higher in advanced cancers). Surgical intervention induced a transient increase in FRAP and TAC with greater FRAP elevation in older, obese patients with several comorbidities. SOD activity significantly increased while GPx non-significantly decreased between 8 and 24 h post-incision. Poorer health status was associated with an increase in SOD and a decrease in GPx at 72 h. Clinical manifestation of postoperative ileus was preceded by decreased TAC at 24 h and an increase in SOD between 8 and 24 h and anastomotic leak was manifested by diminished SOD at 72 h compared to activities at 8 and 24 h. The time-frame between 8 and 24 h post-incision might be the most critical regarding oxidant/antioxidant balance and therefore the best suited for antioxidant-based intervention.

## 1. Introduction

Colorectal cancer (CRC) remains one of the most common types of cancer and the second-leading cause of cancer-related deaths [1] despite considerable progress made in its early diagnostics [2]. Surgical resection of the tumor and the removal of regional lymph nodes continue to be the mainstay of therapy for locoregional CRC [1]. In more advanced stages of disease multi-modality therapy with surgery, chemotherapy, and radiotherapy is often required [3].

Oxidative stress plays a crucial role in carcinogenesis and acts either by promoting or hampering tumor growth depending on the stage of the process [4]. It is believed to induce neoplastic transformation in the colon, especially in patients with inflammatory bowel disease [5]. The incidence rates of CRC are higher in developed countries, a phenomenon attributed to Western lifestyle and diet [1]. Imbalance between dietary prooxidants and antioxidants is a hallmark of Western diets and results from overabundance of saturated fat and refined sugar combined with a shortage of fruits and vegetables [6]. Mechanistically, nutritionally mediated oxidative stress upregulates circulating proinflammatory cytokines such as interleukin (IL)-6 and tumor necrosis factor (TNF)-α in the postprandial period and promotes expression of inflammatory mediators in the longer time-frame [6], increasing the risk of neoplastic transformation. Consistently, exogenous antioxidants, including derivatives of medicinal plants, are intensively explored as potential chemopreventive agents [3]. In turn, the role of endogenous antioxidants in cancer progression is less clear, as various antioxidant enzymes have been shown to promote viability of tumor cells [4]. The repeatedly demonstrated beneficial effects of exogeneous antioxidants as chemotherapeutics, particularly in combination with conventional chemotherapy drugs (reviewed in [3]), have recently been challenged as well (reviewed in [4]).

Surgical intervention causes trauma and evokes immune and inflammatory response, exacerbating cancer-associated oxidative stress and accelerating damage to macromolecules. Resulting local and systemic dysfunction translates into impaired patient recovery [7]. Moreover, surgery-induced oxidative stress is acknowledged to affect oncological outcomes by contributing to local recurrence and the development of secondary tumors following primary tumor resection. Indeed, up to 30% of CRC patients are estimated to develop secondary tumors within five years although no signs of metastasis were present at the time of surgery and disease relapse is noted within the first two years in the vast majority of cases [8].

The perioperative window has been shown to provide conditions promoting growth of residual cancer cells. Yet, it has been referred to as “an underutilized time interval in the treatment strategy of cancer patients” and a role for antioxidant-based targeted therapies has been proposed [8]. However, the effects of clinical trials testing enteral nutrition enriched in antioxidants have been inconsistent [9,10].

Thus, this study was conducted to explore the potential patient-, CRC-, and surgery-related modifiers of serum and erythrocyte antioxidant capacity and to determine the impact of antioxidant status on prevalence of surgical complications and patient recovery. In view of a notion that the magnitude of the trauma affects the degree of oxidative stress [7] and that reduced invasiveness of surgical intervention has an attenuating effect [10], we compared, unexplored before, changes in antioxidant defenses induced in CRC patients by open and robot-assisted approach.

## 2. Materials and Methods

This manuscript was prepared following the strengthening the reporting of observational studies in epidemiology (STROBE) guidelines [11].

### 2.1. Patients

The current study is follow-up research (2019–2021) conducted on biobanked samples collected for the purpose of a prospective non-randomized cohort study investigating inflammatory, immune, and angiogenic response and homeostasis following open and robotic surgery in patients with colorectal cancer and carried out as part of the Wrovasc–Integrated Cardiovascular Centre project (http://www.wssobr-wroc.pl/projekty/wrovasc/ assessed on 22 May 2021).

The study population consisted of consecutive unselected patients admitted to the Department of Surgical Oncology of the Regional Specialist Hospital in Wroclaw, Poland between 2012 and 2015 for the curative resection of histologically confirmed colorectal adenocarcinoma. Exclusion criteria were age <18 years, American Society of Anesthesiologists (ASA) Physical Status Classification system >3, emergency surgery, gross metastatic disease, locally advanced cancers not amenable to curative resection, tumors requiring en bloc multi-visceral resection, and coexisting malignancies. Patients undergoing neoadjuvant chemo- or radiotherapy, receiving immunosuppressants and/or steroids were excluded as well.

Characteristics of the study population included in the current study for antioxidant analysis prior to surgery is presented in Table 1. Characteristics of patients for whom a complete set of samples from all time-points of a follow-up was available is given in Table 2 as patients with missing data at any of time-points were excluded. Patients’ data regarding demographics, anthropometrics, laboratory parameters, comorbidities, perioperative complications, and pathology report, were collected prospectively. Patients’ health was evaluated using the Charlson Comorbidity Score (CCS) in addition to ASA Physical Status Classification system. Robotic surgery was conducted with the assistance of da Vinci^®^ Si surgical system (Intuitive Surgical, Sunnyvale, CA, USA). Cancers were stage pathologically using the tumor–node–metastasis (TNM) system. Patients underwent standardized general anesthesia (induced with propofol, fentanyl, and rocuronium and maintained with sevoflurane). All patients were given metamisol before waking up or immediately after the surgery. Postoperative complications were classified using the Clavien–Dindo classification (CDC) and recorded within 30 days after surgery. Surgical site infections (SSI) were classified in accordance with the Center for Disease Control and Prevention criteria. Restoration of bowel function (RoBF) was defined as the passage of first stool and tolerance of solid diet. Pathological (prolonged) postoperative ileus was defined using a ≥ 5 days threshold [12].

### 2.2. Analysis of Antioxidant Defenses

Antioxidants in serum and erythrocytes were measured in blood sampled in the morning on admission to hospital and at 8, 24, and 72 h post-incision. Collected samples were stored at −80 °C until analysis.

#### 2.2.1. Serum Antioxidants

Venous blood was collected into serum-separator tubes, clotted for 30 min and centrifuged (15 min., 720× *g*, 10 °C). Serum was collected and stored in aliquots to avoid repeated freeze-and-thaw cycles.

##### Total Antioxidant Capacity (TAC)

TAC was measured colorimetrically in duplicates using the Antioxidant Assay Kit from Sigma-Aldrich (St. Louis, MO, USA). The assay is based on the antioxidants’ capacity to suppress the formation of ABTS^•+^, a soluble green radical cation, from ABTS (2,2′-azino-bis(3-ethylbenzthiazoline)-6-sulfonic acid) oxidized by ferryl myoglobin radical formed from metmyoglobin and hydrogen peroxide. Color intensity, inversely related to the antioxidant capacity, was recorded at 405 nm using the microplate spectrophotometer Infinite 200 (Tecan Group Ltd., Männedorf, Switzerland). The standard curve was based on Trolox, a water-soluble vitamin E analog, and data are expressed in Trolox equivalents.

##### Ferric Reducing Antioxidant Power (FRAP)

FRAP was measured colorimetrically in duplicates using Ferric Reducing Antioxidant Power (FRAP) Assay Kit from Sigma-Aldrich. The assay is based on the antioxidants’ capacity to reduce Fe^3+^ to Fe^2+^. The reaction at low pH is accompanied by formation of a colored ferrous–probe complex from a colorless ferric–probe complex. The color intensity, proportional to antioxidant content, was measured at 594 nm using a microplate spectrophotometer Infinite 200 (Tecan Group Ltd.) against ferrous standard.

#### 2.2.2. Erythrocyte Antioxidants

Venous blood was sampled into citrate (anticoagulant)-containing tubes. Erythrocytes were separated from plasma, leukocytes, and platelets by sample centrifugation at 2000× *g* for 15 min at 10 °C, dissolved in cold 0.9% NaCl (1:2, *v*/*v*), and subjected to two cycles of centrifugation (1200× *g* for 5 min at 4 °C) and washing (0.9% NaCl). Erythrocytes were then hemolyzed by dissolving them in cold water (1:1, *v*/*v*) and incubating on ice (15 min).

##### Glutathione Peroxidase (GPx) (EC 1.11.1.9)

The activity of GPx1 was measured spectrophotometrically in erythrocyte hemolysates in duplicates using the Glutathione Peroxidase Assay Kit from Cayman Chemical (Ann Arbor, MI, USA) according to the manufacturer’s instructions. The assay detects the oxidation of NADPH to NADP^+^, recorded at 340 nm, which accompanies reduction of oxidized glutathione by glutathione reductase. Oxidized glutathione is produced by GPx during reduction of hydroperoxide. One unit of enzyme activity is defined as GPx amount resulting in oxidation of 1 μmol of NADPH per minute at 25 °C.

##### Superoxide Dismutase (SOD) (EC 1.15.1.1)

The activity of SOD1 was measured spectrophotometrically in erythrocyte hemolysates in duplicates using the Superoxide Dismutase Assay Kit from Cayman Chemical according to the manufacturer’s instructions. The assay detects superoxide anion generated by xanthine oxidase from xanthine using tetrazolium salt, which is converted to formazan dye recorded colorimetrically at 450 nm. The color intensity is inversely related to SOD1 activity, which converts superoxide anion into hydrogen peroxide. One unit of enzyme activity (U) is defined as the SOD amount needed for 50% inhibition of superoxide formation under the method’s conditions.

### 2.3. Laboratory Paramaters and Inflammatory Cytokines

Data on hemoglobin (HGB) and leukocyte count (WBC), obtained using an automated hematology analyzer, were retrieved from the patient’s medical history.

Previously published data [13] on inflammatory interleukins were retrieved from our database for correlation analysis. They were quantified using a flow cytometry-based method (Luminex xMAP^®^ technology) on the Bio-Plex 200 platform (Bio-Rad, Hercules, CA, USA) and Bio-Plex Pro™ human cytokine, chemokine, and growth factor magnetic bead-based assays.

### 2.4. Statistical Methods

Statistical analysis was completed using MedCalc^®^ Statistical Software version 19.7 (MedCalc Software Ltd., Ostend, Belgium; https://www.medcalc.org; assessed on 22 May 2021). All tests were two-tailed and a *p*-value ≤ 0.05 was considered significant. Data distribution was tested using the Kolmogorov–Smirnov test and homogeneity of variances with the Levene test. Log-transformation was used when appropriate. Data are presented as means of medians with 95% confidence interval (CI). They were analyzed using t-test for independent samples, with Welch correction if appropriate, or Mann–Whitney U test. Multigroup comparisons were conducted using one-way ANOVA or Kruskal–Wallis H test with Student–Newman–Keuls or Conover post-hoc tests, respectively. Correlation analysis was conducted using Spearman rank (ρ) or Pearson (*r*) correlation tests. Analysis of covariance (ANCOVA) or multiple regression were used to co-examine data and identify independent variables. Repeated measures ANOVA and the Friedman test were applied to analyze the dynamics of antioxidants during short follow-up.

## 3. Results

### 3.1. Impact of Patient- and Disease-Related Factors on Preoperative Serum and Erythrocyte Antioxidant Capacity

The possible association between antioxidant capacity and patient-related features (sex, age, BMI, HGB, WBC, general health condition expressed in terms of ASA or CCS score, and inflammatory cytokines IL-1β, IL-6, and TNFα) as well as disease-related factors (histological grade, overall TNM stage, depth of tumor invasion (T), lymph node metastasis (N), and distant metastasis (M)) was evaluated.

#### 3.1.1. Serum Antioxidant Capacity: FRAP and TAC

**Figure 1:**
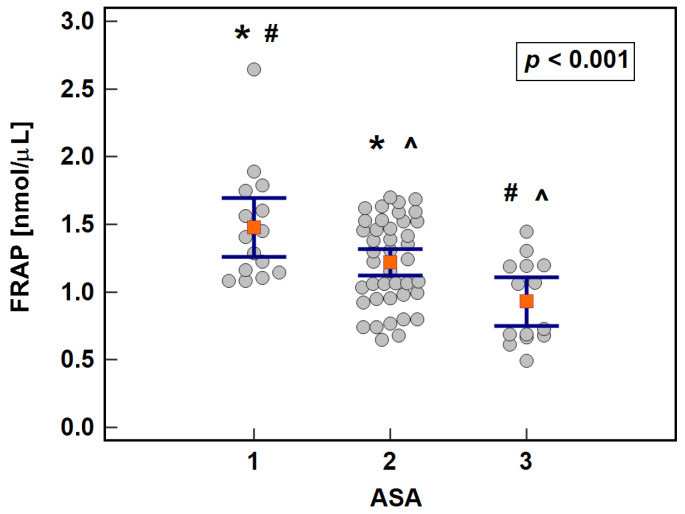
Effect of health condition expressed in terms of ASA score on serum antioxidant capacity measured with FRAP assay in patients with colorectal cancer. Data presented as means with 95% confidence intervals (orange squares with whiskers) and analyzed using one-way ANOVA with Student–Newman–Keuls post-hoc test. Statistically significant differences between groups marked by symbols of the same type (*, #, ^).

Antioxidant capacity measured with FRAP assay was significantly associated with patient-related factors such as age, general health condition, and hemoglobin concentration. It was lower in older patients (*r* = −0.32, *p* = 0.007), in patients with low hemoglobin (*r* = 0.38, *p* = 0.001), or with worse general health as indicated by a negative correlation with CCS (*ρ* = −0.24, *p* = 0.047). The association was more evident when health status was expressed in terms of ASA score (Figure 1).

Multivariate analysis showed that hemoglobin concentration (*p* = 0.014) and ASA status (*p* = 0.008) were significantly associated with FRAP antioxidant capacity when co-examined, while age was not (*p* = 0.160).

Antioxidant capacity measured as TAC was not significantly associated with any patient-related factors but negatively correlated with concentrations of inflammatory mediators: IL-1β (*r* = −0.42, *p* < 0.001), IL-6 (*r* = −0.37, *p* = 0.002), and TNFα (*r* = −0.43, *p* < 0.001).

In multivariate analysis, TNFα was selected as an independent predictor of TAC level with partial correlation coefficient (*r*_p_) = −0.49, *p* < 0.001.

Antioxidant capacity, measured either by FRAP or TAC assay, was not significantly associated with cancer pathology.

#### 3.1.2. Erythrocyte Antioxidants: SOD1 and GPx1

The GPx activity in erythrocytes was negatively correlated with cancer stage (*ρ* = −0.37, *p* = 0.005) and histological grade (*ρ* = −0.48, *p* < 0.001) and was non-significantly lower in patients with distant metastasis (3.97 μmol/min/mL (3.5–4.44) in M0 vs. 2.64 μmol/min/mL (0.57–4.71) in M1, *p* = 0.072). It was not associated with patient-related factors and did not correlate with inflammatory mediators.

The SOD activity in erythrocytes was positively correlated with cancer stage (*ρ* = 0.34, *p* = 0.012) and inflammatory mediators IL-1β (*r* = 0.38, *p* = 0.006) and TNFα (*r* = 0.42, *p* = 0.002) but was not associated with patient-related features.

### 3.2. Impact of Patient- and Disease-Related Factors on Serum Antioxidant Capacity and Erythrocyte Antioxidant Enzymes during Early Postoperative Period

Changes in antioxidant potential during early postoperative period were determined at 8, 24, and 72 h post-incision and the potential effect of patient- and disease-related factors on their time-course was assessed. For the purpose of analysis, continuous variables such as age, BMI, and HGB were dichotomized using the following criteria: ≥75 yrs., >25 kg/m^2^, and <120 g/L (females) or <130 g/L (males). Categorical data were grouped in the following manner: ASA1 and ASA2 against ASA3; G1 and G2 against G3 and G4; TNM stage 0, I, and II against III and IV; tis, T1, and T2 against T3 and T4; and N0 against N1 and N2.

The possible effect of surgery-related factors such as type of surgery (open vs. robotic), EBL (≥150 mL), transfusions, length of surgery (LoS; ≥175 min), and number of excised lymph nodes (≥15) was evaluated as well.

In addition, the potential association of antioxidant capacity with adverse clinical outcomes such as anastomotic leak, delayed restoration of bowel function (≥5 days), SSI, and prolonged hospitalization (≥6 days) was investigated.

#### 3.2.1. Serum Antioxidant Capacity: FRAP and TAC

**Figure 2:**
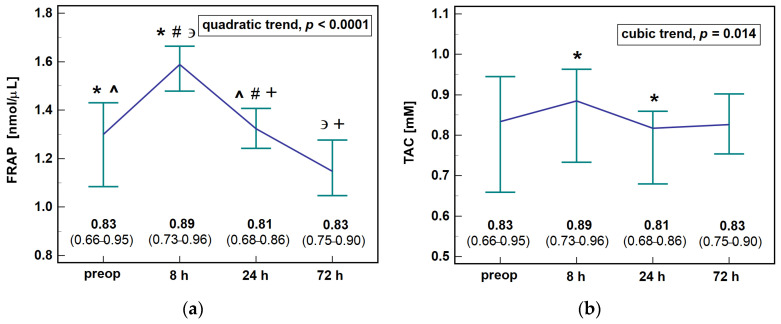
The level of serum antioxidant capacity in colorectal cancer patients during early postoperative period: (**a**) FRAP and (**b**) TAC. Data presented as medians with 95% confidence interval and analyzed using Friedman test. Significant (*p* < 0.05) differences between individual time-points are indicated by symbols of the same type (*, ^, #, +, ∍). Preop, preoperative; 8 h, 8 h post-incision; 24 h, 24 h post-incision; 72 h, 72 h post-incision. Of the modifiers evaluated using two-factor repeated measures ANOVA, the dynamics of FRAP were affected by the patient’s age (Figure 3a), health status (Figure 3b), and body mass index (Figure 3c). Regarding age, higher initial increase was observed in older than younger patients (Δ_8h/0_: 1.4-fold vs. 1.1-fold, *p* = 0.015). FRAP increased after 24 h as compared to baseline (Δ_24h/0_) in ASA3 and not ASA1/2 patients (1.5-fold vs. 1.0, *p* = 0.008). Regarding BMI, higher initial increase was found in overweight/obese patients than those with BMI < 25 (Δ_8h/0_: 1.4-fold vs. 1.1-fold, *p* = 0.018). The detailed results for all analyzed factors with significance of between- and within-subject effects and their interaction are presented in Supplementary Materials, Table S1.

Antioxidant capacity of the serum in the early postoperative period initially increased but dropped after 8 h post-incision. It displayed more expressive dynamics with significant differences between individual time-points when determined with FRAP assay (Figure 2a) while TAC assay showed a significant drop solely between 8 and 24 h post-incision (Figure 2b).

**Figure 4:**
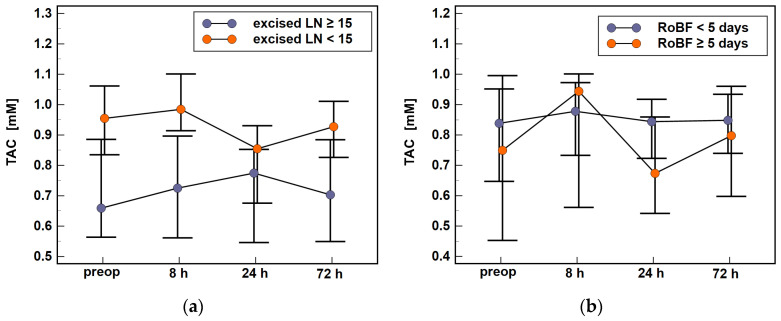
Association between the time-course of TAC and clinical data: (**a**) extent of surgery and (**b**) postoperative ileus. Data analyzed using two-factor repeated measures ANOVA following log-transformation and presented as medians with 95% confidence interval. Preop, preoperative; 8 h, 8 h post-incision; 24 h, 24 h post-incision; 72 h, 72 h post-incision; LN, lymph nodes; RoBF, restoration of bowel function.

The dynamics of TAC was affected by none of the evaluated modifiers. However, the extent of surgery expressed in terms of the number of excised lymph nodes had a significant impact on TAC preoperatively (0.66 mM vs. 0.96 mM, *p* = 0.015) and at 8 h (0.73 mM vs. 0.99 mM, *p* = 0.003) and 72 h post-incision (0.70 mM vs. 0.93 mM, *p* = 0.009), as patients with higher number of excised lymph nodes had lower TAC levels (Figure 4a). Of the evaluated clinical outcomes, pathological postoperative ileus was associated with significantly lower TAC at 24 h post-incision (0.55 mM vs. 0.76 mM, *p* = 0.038) (Figure 4b).

The detailed results for all analyzed factors with significance of between- and within-subject effects and their interaction are presented in Supplementary Materials, Table S2.

There was no significant correlation between FRAP and TAC at any given time-point during a follow-up.

#### 3.2.2. Erythrocyte Antioxidants: SOD and GPx

**Figure 5:**
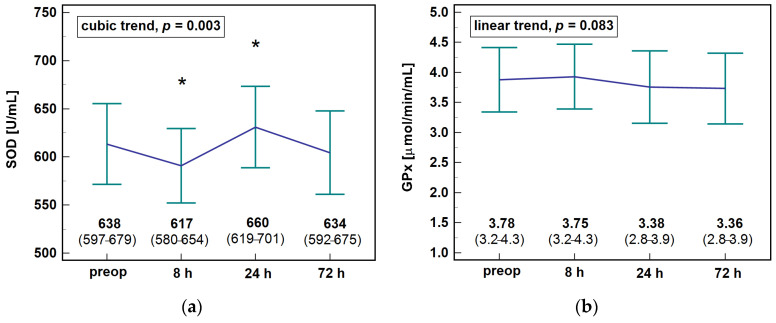
The activity of erythrocyte antioxidants in colorectal cancer patients during the early postoperative period: (**a**) superoxide anion dismutase (SOD) and (**b**) glutathione peroxidase (GPx). Data presented as means with 95% confidence interval and analyzed using ANOVA on repeated measures. Significant (*p* < 0.05) differences between individual time-points are indicated by asterisks (*). Preop, preoperative; 8 h, 8 h post-incision; 24 h, 24 h post-incision; 72 h, 72 h post-incision.

The erythrocyte SOD and GPx displayed different patterns during the early postoperative period, with SOD activities significantly increasing between 8 and 24 h post-incision and GPx activities non-significantly declining (Figure 5).

**Figure 6:**
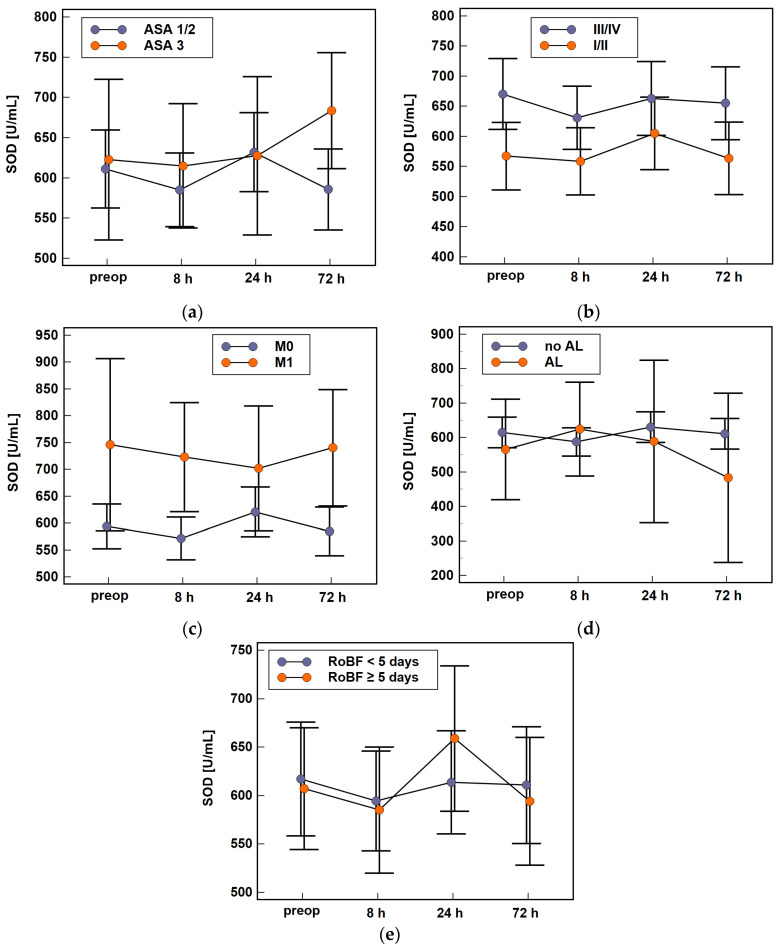
The association of SOD time-course with patient- and disease-related factors and clinical outcomes: (**a**) the American Society of Anesthesiologists (ASA) physical status classification system; (**b**) cancer TNM stage; (**c**) presence of distant metastases (stage M); (**d**) anastomotic leak (AL); and (**e**) delayed restoration of bowel function (RoBF). Data analyzed using two-factor repeated measures ANOVA and presented as means with 95% confidence interval. Preop, preoperative; 8 h, 8 h post-incision; 24 h, 24 h post-incision; 72 h, 72 h post-incision.

Of the evaluated modifiers, the activity of SOD was affected by patient’s health status (Figure 6a) and cancer stage (Figure 6b), particularly the presence of distant metastases (Figure 6c). The enzyme activity was also associated with clinical outcomes, that is, the presence of anastomotic leak (Figure 6d) and postoperative ileus (Figure 6e).

Of those, ASA and AL were associated with the time-course of SOD activity. It slightly dropped in ASA1/2 patients but increased in ASA3 patients at 72 h as compared to 24 h (Δ_72h/24h_: 0.9-fold vs. 1.1-fold, *p* = 0.001) and decreased at 72 h as compared to 24 h and 8 h in patients with AL, while being at the same level in patients without AL (Δ_72h/24h_: 0.8-fold vs. 1, *p* = 0.049 and Δ_72h/8h_: 0.8-fold vs. 1, *p* = 0.042). Moreover, SOD activity between 8 h and 24 h increased in patients with postoperative ileus (Δ_24h/8h_: 1.1-fold vs. 1, *p* = 0.037).

Cancer stage and presence of metastases affected SOD activity at given time-points but had no impact on enzyme dynamics. Patients with advanced cancers (stages III and IV) had higher SOD activities than those with stage 0, I, and II cancers, significantly so at baseline (670 U/mL vs. 567 U/mL, *p* = 0.012) and at 72 h (655 U/mL vs. 564 U/mL, *p* = 0.033). Patients with M1 had higher SOD activity preoperatively (746 U/mL vs. 594 U/mL, *p* = 0.013) and at 8 h (723 U/mL vs. 572 U/mL, *p* = 0.007) and 72 h (740 U/mL vs. 585 U/mL, *p* = 0.014).

The detailed results for all analyzed factors with significance of between- and within-subject effects and their interaction are presented in Supplementary Materials, Table S3.

**Figure 7:**
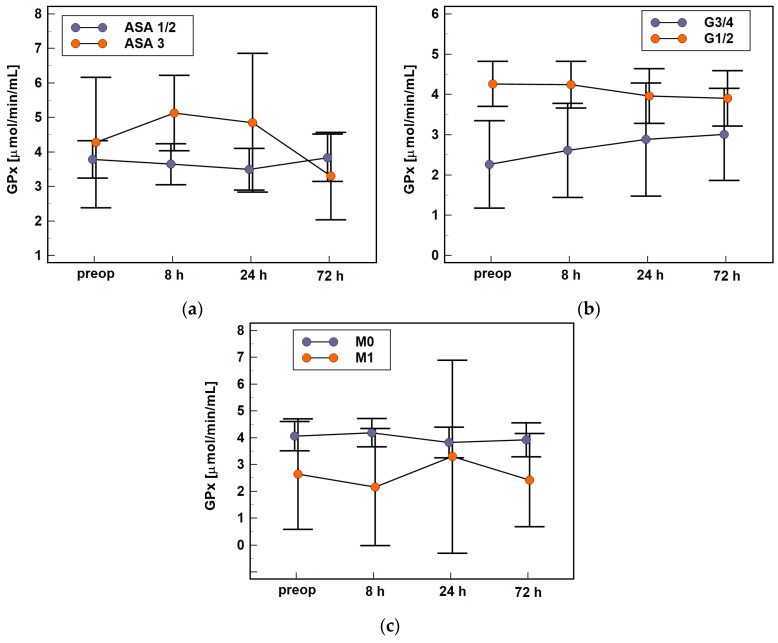
Effect of patient- and disease-related factors on GPx time-course: (**a**) the American Society of Anesthesiologists (ASA) physical status classification system; (**b**) tumor histological grade (G); and (**c**) distant metastases (stage M). Data analyzed using two-factor repeated measures ANOVA and presented as means with 95% confidence interval. Preop, preoperative; 8 h, 8 h post-incision; 24 h, 24 h post-incision; 72 h, 72 h post-incision.

Of the analyzed modifiers, GPx time-course was affected by health status (Figure 7a), with an initial rise in enzyme activity observed solely in ASA3 patients (Δ_8h/0_: 1.4-fold vs. 1, *p* = 0.032). Higher tumor aggressiveness (G3 and G4) was associated with lower GPx activity than lower aggressiveness (G1 and G2), both preoperatively (2.26 vs. 4.26 μmol/min/mL, *p* = 0.002) and at 8 h (2.61 vs. 4.24 μmol/min/mL, *p* = 0.015). The differences at other time-points were non-significant because of different GPx dynamics—the enzyme activity decreased during a follow-up in patients with less aggressive tumors and increased in those with more aggressive tumors (Figure 7b). Consequently, enzyme activity at 72 h as compared to baseline (Δ_72h/0_) was higher in patients with G3/4 than G1/2 tumors (1.3-fold vs. 0.85-fold, *p* = 0.017). GPx activity was lower in M1 than M0 patients (Figure 7c), significantly so at 8 h post-incision (2.16 vs. 4.19 μmol/min/mL, *p* = 0.010).

The detailed results for all analyzed factors with significance of between- and within-subject effects and their interaction are presented in Supplementary Materials, Table S4.

**Figure 8:**
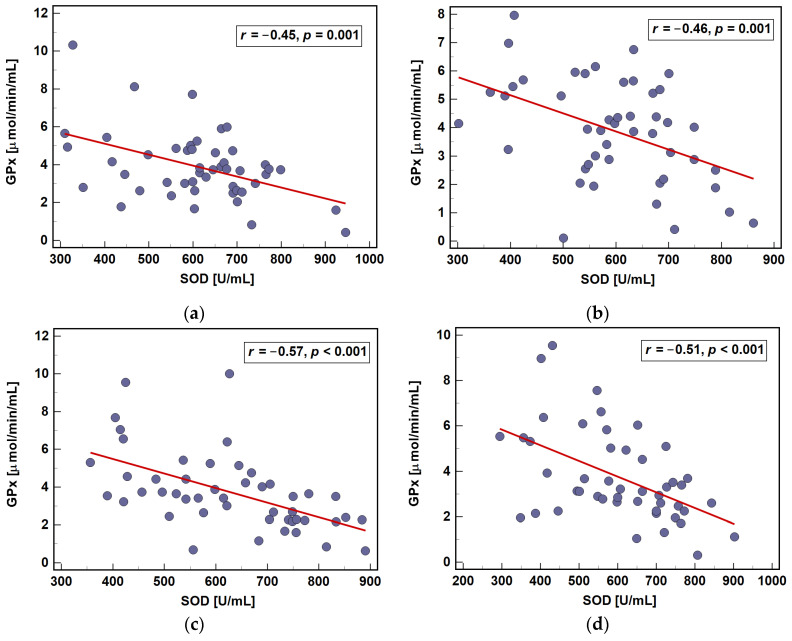
Interrelationship between SOD and GPx activities during perioperative period: (**a**) prior operation; (**b**) at 8 h post-incision; (**c**) at 24 h post-incision; and (**d**) at 72 h post-incision. Data analyzed using Pearson correlation test and presented as correlation coefficient (*r*).

The activities of SOD and GPx were inversely related to each other at any given time-point (Figure 8).

## 4. Discussion

A better understanding of antioxidant status, its dynamics in the early postoperative period, and association with clinical outcomes as well as an identification of potential modifiers is needed for successful targeted antioxidant therapies [7]. Analysis of antioxidant status at baseline demonstrated that older or obese patients or those with accumulated comorbidities (mostly type 2 diabetes and cardiovascular diseases) had lower plasma antioxidant capacity, corroborating an established link between ageing or cardiometabolic disorders and oxidative stress [14,15]. Consistently, TAC showed an inverse relationship with inflammation, which accompanies these conditions [16]. Antioxidant capacity was directly and independently associated with hemoglobin concentration, supporting findings in other cancer types [17,18]. This observation is also consistent with oxidative stress being involved in the development of anemia of chronic diseases [18,19], which accompanies cancer and is associated with its aggressiveness [20,21].

Erythrocyte antioxidants reflected the disease advancement with GPx being lower and SOD higher in advanced CRC. Corroborating findings in breast cancer [22], GPx negatively correlated with tumor aggressiveness. SOD yields hydrogen peroxide, therefore, its enhanced activity is disadvantageous if not coupled with GPx or catalase. Indeed, oxidative-stress-related conditions are often characterized by upregulated SOD and downregulated GPx [23]. Highlighting the negative aspects of SOD upregulation, its activity in our patients was positively correlated with inflammatory mediators and inversely with GPx.

Markedly diminished preoperative antioxidant capacity in elderly and in metabolically disturbed patients is worrisome as surgical procedure only deepens oxidative stress [7]. Moreover, the incidence of cardiometabolic diseases is increasing [24] as is the age of patients undergoing colorectal surgery [25]. Indeed, major gastrointestinal surgery results in a drop in circulating antioxidants [9] and surgery in geriatric CRC patients, in poorer health condition, is accompanied by longer hospitalization, increased risk of complications, and higher mortality [26]. Surprisingly, however, surgery in our patients induced a transient elevation in plasma antioxidant capacity eight hours post-incision, accompanied by downregulation of SOD activity in erythrocytes. The likely explanation is the contribution of applied anesthetics, known to possess antioxidant properties. As oxidant/antioxidant balance is affected by type and duration of anesthesia [7], the same protocol was applied to all our patients. They were treated with propofol, fentanyl, and rocuronium for induction and the general anesthesia was maintained with sevoflurane. While intravenous analgesics are claimed to scavenge reactive oxygen species, volatile anesthetics may, reportedly, induce oxidative stress during longer procedures (reviewed in [27]). The length of surgery and therefore the time our patients stayed under anesthesia differed but none of the evaluated antioxidants correlated with length of surgery. Free radical-scavenging properties or indirect antioxidant activity have been reported for propofol [27,28,29,30,31], fentanyl [29,32], and sevoflurane [33] as well as for rocuronium [34,35] muscle relaxant, likely contributing to an initial increase in antioxidant capacity.

However, detailed analysis showed that FRAP levels increased solely in elderly or obese patients or with accumulated comorbidities. In fact, ASA = 3 patients also experienced transient elevation in erythrocyte GPx. Therefore, the precise conditions responsible for lower antioxidant capacity at baseline were associated with improved response following surgery. This counterintuitive observation can be mostly explained by differences in the pharmacodynamics of applied anesthetic drugs. Propofol and opioid doses are adjusted to body mass [36], thus overweight/obese patients received more anesthesia and may have more elevated FRAP as a consequence. Moreover, propofol concentration in plasma is generally higher in obese than lean individuals owing to blood preferentially distributing the drug to non-adipose tissue [37]. Furthermore, cardiometabolic diseases and age are associated with impaired hepatic metabolism [38] and disturbed renal excretion, causing decreased clearance of anesthetic drugs [36,37]. In addition, a more pronounced activation of NFkB in response to surgical trauma may occur in elderly patients or those with cardiometabolic diseases [39]. It controls expression of antioxidant enzymes [40,41] and proinflammatory cytokines. The resulting overabundance of the latter is likely responsible for the transient nature of the phenomenon as antioxidant enzymes are sensitive to inflammation and oxidative stress [40].

The magnitude of trauma is directly correlated with severity of oxidative stress [7] and transfusions have been shown to affect antioxidant capacity at 7 and 14 days [42]. In the early postoperative period, however, neither blood loss nor the number of excised lymph nodes affected antioxidant dynamics. Still, the more lymph nodes that were excised, the lower the TAC level observed in patients at any investigated time-point. As TAC was also lower preoperatively, the association may not reflect the impact of surgery extent on antioxidant capacity and needs further clarification. In particular, there was no significant difference in serum or erythrocyte antioxidants between patients undergoing open and minimally invasive robotic surgery. While there are no previous reports regarding the impact of robotic surgery in CRC patients on antioxidants, an inflammatory response has been shown to be reduced [13,43,44]. As longer exposure to volatile anesthetics induces oxidative stress [27], prolonged exposure to sevoflurane during robotic surgery might counteract the beneficial effect of minimizing the invasiveness of surgery.

A link between oxidative stress and adverse clinical outcomes following surgery has been established. The perioperative administration of antioxidants and thus alleviation of oxidative stress is considered an alternative approach to improving outcomes (reviewed in [7]). Here, we found that the period between 8 and 24 h post-incision might be the most critical regarding oxidant/antioxidant balance as there is a significant rise of erythrocyte SOD activity, which is paired neither with increased GPx nor serum antioxidant capacity, which is dropping due to the antioxidant effect of anesthesia wearing off. Therefore, the time-frame directly after surgery seems to be optimal for starting intervention based on antioxidants. Regarding clinical outcomes, we sought the link between serum and erythrocyte antioxidant capacity and presence of anastomotic leak, postoperative pathological ileus, surgical site infections, and prolonged hospitalization. Of these, TAC and SOD were significantly related to the postoperative ileus as the restoration of bowel function was delayed beyond the 5th day in patients who had lower TAC levels at 24 h post-incision or those that experienced an increase in SOD activity between 8 and 24 h. These observations correspond well with the pathogenic role of inflammation and oxidative stress in impairing gastrointestinal motility in response to surgical trauma [45]. Additionally, SOD dynamics differed in patients with AL, the most serious of complications. Preceding its clinical manifestation, AL patients experienced an initial rise in SOD followed by decrease in enzyme activity while relatively stable enzyme activities were observed in non-AL patients.

In our research we used two different methodologies of assessing so-called total antioxidant capacity, FRAP, and TAC, as they are characterized by varying degrees of reactivity towards individual serum/plasma antioxidants [46], explaining the lack of correlation between their results.

The limitations of this study are that it was a follow-up on research and used already-collected samples and that the original study was conducted on a relatively small cohort that lacked randomization between patients undergoing robotic or open surgery. However, during the study period, robotic surgery in Europe was still considered a novel (almost experimental) approach which made randomization difficult. As for analytical methods, information on erythrocyte antioxidant capacity would be more complete if it included determination of catalase activity.

## 5. Conclusions

Cancer pathology, patient age, and comorbidities are associated with diminished systemic antioxidant defense in colorectal cancer and alter antioxidant dynamics during the early postoperative period pointing to elderly patients with several comorbidities and/or with advanced cancer as those who may especially benefit from antioxidant therapy. The period between 8 and 24 h post-incision seems to be the most critical regarding oxidant/antioxidant balance as there is a significant rise of erythrocyte SOD activity, which is not paired with increased GPx. Likely to further exacerbate the oxidative stress, non-counteracted SOD activity is accompanied by a significant decrease in serum antioxidant capacity, regardless of whether evaluated using the FRAP or ABTS (TAC) method. Therefore, the time-frame directly after surgery might be optimal for starting intervention based on antioxidants. Considering TAC and SOD association with RoBF, such interventions might prevent pathological postoperative ileus and thus facilitate convalescence and reduce postoperative morbidity.

## Figures and Tables

**Figure 3:**
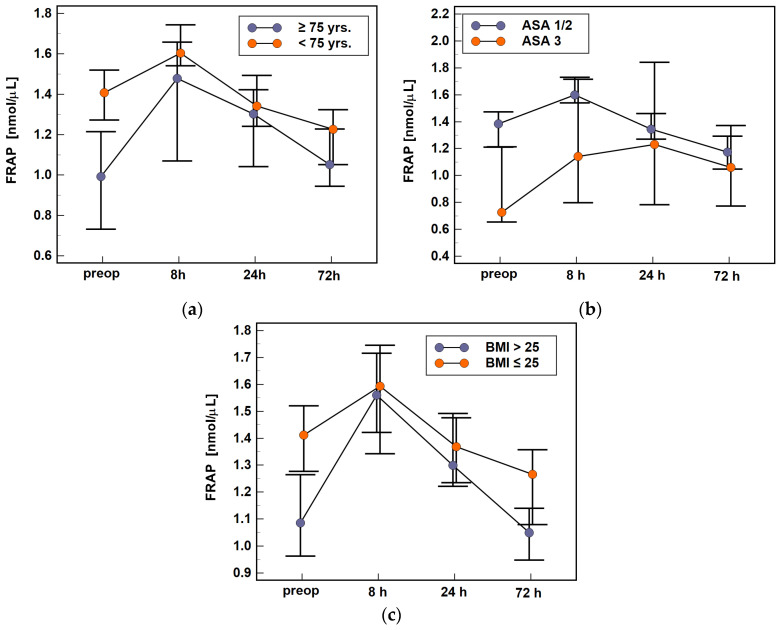
Effect of patient-related factors on FRAP time-course: (**a**) age; (**b**) the American Society of Anesthesiologists (ASA) physical status classification system; and (**c**) body mass index (BMI). Data analyzed using two-factor repeated measures ANOVA following log-transformation and presented as medians with 95% confidence interval. Preop, preoperative; 8 h, 8 h post-incision; 24 h, 24 h post-incision; 72 h, 72 h post-incision.

**Table 1:** Characteristics of patients included in the analysis of serum and erythrocyte antioxidant defense prior to surgery.

Characteristics	Serum AOXs	Erythrocyte AOXs
*N*	72	58
Demographics and anthropometrics:		
Sex, F/M (*n*)	28/44	22/36
Age (y), mean (95% CI)	67.2 (64.7–69.7)	65.8 (62.9–68.8)
BMI (kg/m^2^), mean (95% CI)	26.15 (24.8–27.6)	27.04 (25.8–28.3)
Health status:		
ASA, 1/2/3 (*n*)	15/46/11	12/36/10
CCS, median (range)	5 (2–8)	4 (2–7)
WBC (×10^9^/L), mean (95% CI)	7.02 (6.51–7.52)	6.88 (6.37–7.38)
HGB (g/dL), mean (95% CI)	12.06 (11.59–12.53)	12.27 (11.77–12.78)
IL-1β (pg/mL), mean (95% CI)	1.96 (1.72–2.24)	1.79 (1.52–2.1)
IL-6 (pg/mL), mean (95% CI)	14.2 (12.06–16.72)	14.3 (11.72–17.45)
TNFα (pg/mL), mean (95% CI)	32.64 (29.01–36.71)	30.5 (26.49–35.11)
Oncological features:		
TNM, 0-I/II/III/IV (*n*)	11/29/27/5	10/21/21/6
T, Tis/1/2/3/4 (*n*)	5/1/8/43/15	4/2/6/37/9
N, 0/1/2 (*n*)	38/18/16	31/13/14
M, 0/1 (*n*)	67/5	52/6
G, 1/2/3/4/x (*n*)	12/48/10/1/1	9/39/7/3

AOXs, antioxidants; *N*, number of observations; F, females; M, males; y, years; CI, confidence interval; BMI, body mass index; ASA, the American Society of Anesthesiologists Physical Status Classification System; CCS, the Charlson Comorbidity Score; WBC, white blood cells; HGB, hemoglobin; IL, interleukin; TNF, tumor necrosis factor; TNM, tumor–node–metastasis staging system; G, histopathological grade.

**Table 2:** Characteristics of patients included in the analysis of serum and erythrocyte antioxidant defense during perioperative period.

Characteristics	Serum AOXs	Erythrocyte AOXs
*N*	59	47
Demographics:		
Sex, F/M (*n*)	20/39	16/31
Age (y), mean (95% CI)	67.1 (64.2–70.1)	65.0 (61.7–68.2)
BMI [kg/m^2^], mean (95% CI)	26.0 (24.7–27.37)	26.74 (25.42–28.06)
ASA, 1/2/3 (*n*)	11/39/9	13/29/5
CCS, median (range)	5 (2–8)	4 (2–7)
Oncological features:		
TNM, 0-I/II/III/IV (*n*)	9/26/19/5	8/18/15/6
T, Tis/1/2/3/4 (*n*)	4/1/6/35/13	3/1/6/29/8
N, 0/1/2 (*n*)	33/13/13	26/10/11
M, 0/1 (*n*)	54/5	41/6
G, 1/2/3/4/x (*n*)	11/37/9/1/1	6/32/6/3
Surgical features:		
Type, open/robotic (*n*)	29/30	25/22
Procedure, APR/LH/LAR/RH/SR (*n*)	2/4/22/18/13	1/3/21/11/11
Length of surgery (min), median (range)	165 (50–360)	165 (150–203)
EBL (mL), median (range)	100 (30–300)	125 (100–200)
Harvested LN, median (range)	14 (3–43)	14 (3–43)
CDC (≥3), no/yes (*n*)	56/3	44/3
SSI, no/yes (*n*)	47/12	38/9
RoBF (≥5 days), no/yes (*n*)	38/21	29/18
LoHS (days), median (range)	6 (4–20)	6 (3–20)

AOXs, antioxidants; *N*, number of observations; F, females; M, males; y, years; CI, confidence interval; BMI, body mass index; ASA, the American Society of Anesthesiologists Physical Status Classification System; CCS, the Charlson Comorbidity Score; TNM, tumor–node–metastasis staging system; G, histopathological grade; APR, abdominal perineal resection; LH, left hemicolectomy; LAR, low anterior resection; RH, right hemicolectomy; SR, sigmoid resection; EBL, estimated blood loss; LN, lymph nodes; CDC, the Clavien–Dindo classification of postoperative complications; SSI, surgical site infections; RoBF, restoration of bowel function; LoHS, length of hospital stay.

## Data Availability

Data is contained within the article or supplementary material.

## Supplementary Materials

The following are available online at https://www.mdpi.com/article/10.3390/antiox10070999/s1: Table S1: Effect of patient-, disease-, and surgery-related factors on FRAP time-course and the association with adverse clinical outcomes—results of two-factor repeated measures ANOVA. Table S2: Effect of patients, disease-, and surgery-related factors on TAC time-course and the association with adverse clinical outcomes—results of two-factor repeated measures ANOVA. Table S3: Effect of patient-, disease-, and surgery-related factors on SOD time-course and the association with adverse clinical outcomes—results of two-factor repeated measures ANOVA. Table S4: Effect of patient-, disease-, and surgery-related factors on GPx time-course and the association with adverse clinical outcomes—results of two-factor repeated measures ANOVA.
