# Supplementary material for: Serum and Erythrocyte Antioxidant Defense in Colorectal Cancer Patients during Early Postoperative Period: Potential Modifiers and Impact on Clinical Outcomes
Source: Antioxidants (Basel). 2021 Jun 23;10(7):999. doi: 10.3390/antiox10070999 (PMC8300787; doi:10.3390/antiox10070999)
Supplement: Supplementary file 1 [file antioxidants-10-00999-s001.zip › antioxidants-1253039-supplementary.pdf]

Supplementary material

Table S1. Effect of patients-, disease-, surgery-related factors on FRAP time-course and the association with adverse clinical outcomes – results of two-factor repeated measures ANOVA.

Group	Group effect	Time effect	Group × time interaction effect
**Patient**			
Sex (F/M)	p = 0.279	p < 0.001	p = 0.982
Age [1]	p = 0.012	p < 0.001	p = 0.116
Obesity [2]	p = 0.462	p < 0.001	p = 0.054
Anemia [3]	p = 0.332	p < 0.001	p = 0.528
ASA [4]	p = 0.009	p < 0.001	p = 0.004
**Disease**			
G [5]	p = 0.787	p = 0.004	p = 0.147
M (n/y)	p = 0.197	p = 0.008	p = 0.776
N [6]	p = 0.288	p < 0.001	p = 0.390
T [7]	p = 0.735	p < 0.001	p = 0.530
TNM stage [8]	p = 0.288	p < 0.001	p = 0.390
**Surgery**			
type [9]	p = 0.888	p < 0.001	p = 0.284
EBL [10]	p = 0.607	p < 0.001	p = 0.275
Transfusions (n/y)	p = 0.123	p = 0.003	p = 0.418
Length of surgery [11]	p = 0.712	p < 0.001	p = 0.233
Excised LN [12]	p = 0.280	p < 0.001	p = 0.636
**Adverse clinical outcomes**			
AL (n/y)	p = 0.309	p = 0.104	p = 0.784
RoBF [13]	p = 0.171	p < 0.001	p = 0.971
SSI (n/y)	p = 0.686	p < 0.001	p = 0.735
LoHS [14]	p = 0.126	p < 0.001	p = 0.544

[1] Dichotomized using ≥75 yrs. as a cut-off; [2] dichotomized using ≥25 kg/m^2 as a cut-off; [3] dichotomized using 120 g/L or 130 g/L cut-offs for females and males, respectively; [4] grouped into ASA=1/2 and ASA=3; [5] grouped into G=1/2 and G=3/4; [6] grouped into N0 and N1/2; [7] grouped into tis/T1/T2 and T3/4; [8] grouped into stage 0/I/II and III/IV; [9] open and robotic; [10] dichotomized using ≥150 ml as a cut-off; [11] dichotomized using ≥175 minutes as a cut-off; [12] dichotomized using ≥15 as a cut-off; [13] dichotomized using ≥5 days as a cut-off; [14] dichotomized using ≥6 days as a cut-off. F, females; M, males; ASA, the American Society of Anesthesiologists physical status classification system; G, histological grade; n/y, no/yes; M, distant metastasis; N, lymph node metastasis; T, depth of tumor invasion; EBL, estimated blood loss; LN, lymph nodes; AL, anastomotic leak; RoBF, restoration of bowel function; SSI, surgical site infections; LoHS, length of hospital stay.

Table S2. Effect of patients-, disease-, surgery-related factors on TAC time-course and the association with adverse clinical outcomes – results of two-factor repeated measures ANOVA.

Group	Group effect	Time effect	Group × time interaction effect
**Patient**			
Sex (F/M)	p = 0.242	p = 0.369	p = 0.587
Age [1]	p = 0.884	p = 0.212	p = 0.262
Obesity [2]	p = 0.265	p = 0.673	p = 0.100
Anemia [3]	p = 0.487	p = 0.339	p = 0.570
ASA [4]	p = 0.355	p = 0.765	p = 0.777
**Disease**			
G [5]	p = 0.744	p = 0.942	p = 0.480
M (n/y)	p = 0.710	p = 0.959	p = 0.869
N [6]	p = 0.548	p =0.399	p = 0.824
T [7]	p = 0.456	p = 0.852	p = 0.821
TNM stage [8]	p = 0.548	p = 0.399	p = 0.824
**Surgery**			
type [9]	p = 0.344	p = 0.406	p = 0.419
EBL [10]	p = 0.114	p = 0.387	p = 0.477
Transfusions (n/y)	p = 0.319	p = 0.751	p = 0.995
Length of surgery [11]	p = 0.398	p = 0.386	p = 0.730
Excised LN [12]	p = 0.030	p = 0.450	p = 0.555
**Adverse clinical outcomes**			
AL (n/y)	p = 0.807	p = 0.968	p = 0.953
RoBF [13]	p = 0.053	p = 0.271	p = 0.623
SSI (n/y)	p = 0.663	p = 0.964	p = 0.206
LoHS [14]	p = 0.171	p = 0.368	p = 0.593

[1] Dichotomized using ≥75 yrs. as a cut-off; [2] dichotomized using ≥25 kg/m^2 as a cut-off; [3] dichotomized using 120 g/L or 130 g/L cut-offs for females and males, respectively; [4] grouped into ASA=1/2 and ASA=3; [5] grouped into G=1/2 and G=3/4; [6] grouped into N0 and N1/2; [7] grouped into tis/T1/T2 and T3/4; [8] grouped into stage 0/I/II and III/IV; [9] open and robotic; [10] dichotomized using ≥150 ml as a cut-off; [11] dichotomized using ≥175 minutes as a cut-off; [12] dichotomized using ≥15 as a cut-off; [13] dichotomized using ≥5 days as a cut-off; [14] dichotomized using ≥6 days as a cut-off. F, females; M, males; ASA, the American Society of Anesthesiologists physical status classification system; G, histological grade; n/y, no/yes; M, distant metastasis; N, lymph node metastasis; T, depth of tumor invasion; EBL, estimated blood loss; LN, lymph nodes; AL, anastomotic leak; RoBF, restoration of bowel function; SSI, surgical site infections; LoHS, length of hospital stay.

Table S3. Effect of patients-, disease-, surgery-related factors on SOD time-course and the association with adverse clinical outcomes – results of two-factor repeated measures ANOVA.

Group	Group effect	Time effect	Group × time interaction effect
**Patient**			
Sex (F/M)	p = 0.698	p = 0.037	p = 0.903
Age [1]	p = 0.132	p = 0.127	P = 0.373
Obesity [2]	p = 0.246	p = 0.054	p = 0.659
Anemia [3]	p = 0.875	p = 0.023	p = 0.482
ASA [4]	p = 0.489	p = 0.186	p = 0.018
**Disease**			
G [5]	p = 0.257	p = 0.454	p = 0.478
M (n/y)	p = 0.015	p = 0.733	p = 0.209
N [6]	p = 0.387	p = 0.121	p = 0.148
T [7]	p = 0.735	p = 0.028	p = 0.113
TNM stage [8]	p = 0.031	p = 0.040	p = 0.382
**Surgery**			
type [9]	p = 0.655	p = 0.038	p = 0.438
EBL [10]	p = 0.695	p = 0.034	p = 0.784
Transfusions (n/y)	p = 0.128	p = 0.165	p = 0.480
Length of surgery [11]	p = 0.884	p = 0.037	p = 0.609
Excised LN [12]	p = 0.433	p = 0.045	p = 0.914
**Adverse clinical outcomes**			
AL (n/y)	p = 0.865	p = 0.059	p = 0.049
RoBF [13]	p = 0.955	p = 0.010	p = 0.101
SSI (n/y)	p = 0.244	p = 0.463	p = 0.694
LoHS [14]	p = 0.637	p = 0.035	p = 0.484

[1] Dichotomized using ≥75 yrs. as a cut-off; [2] dichotomized using ≥25 kg/m^2 as a cut-off; [3] dichotomized using 120 g/L or 130 g/L cut-offs for females and males, respectively; [4] grouped into ASA=1/2 and ASA=3; [5] grouped into G=1/2 and G=3/4; [6] grouped into N0 and N1/2; [7] grouped into tis/T1/T2 and T3/4; [8] grouped into stage 0/I/II and III/IV; [9] open and robotic; [10] dichotomized using ≥150 ml as a cut-off; [11] dichotomized using ≥175 minutes as a cut-off; [12] dichotomized using ≥15 as a cut-off; [13] dichotomized using ≥5 days as a cut-off; [14] dichotomized using ≥6 days as a cut-off. F, females; M, males; ASA, the American Society of Anesthesiologists physical status classification system; G, histological grade; n/y, no/yes; M, distant metastasis; N, lymph node metastasis; T, depth of tumor invasion; EBL, estimated blood loss; LN, lymph nodes; AL, anastomotic leak; RoBF, restoration of bowel function; SSI, surgical site infections; LoHS, length of hospital stay.

Table S4. Effect of patients-, disease-, surgery-related factors on GPx time-course and the association with adverse clinical outcomes – results of two-factor repeated measures ANOVA.

Group	Group effect	Time effect	Group × time interaction effect
**Patient**			
Sex (F/M)	p = 0.329	p = 0.811	p = 0.922
Age [1]	p = 0.096	p = 0.311	p = 0.161
Obesity [2]	p = 0.470	p = 0.749	p = 0.586
Anemia [3]	p = 0.820	p = 0.976	p = 0.725
ASA [4]	p = 0.224	p = 0.112	p = 0.014
**Disease**			
G [5]	p = 0.012	p = 0.946	p = 0.385
M (n/y)	p = 0.040	p = 0.761	p = 0.342
N [6]	p = 0.937	p = 0.836	p = 0.354
T [7]	p = 0.640	p = 0.953	p = 0.876
TNM stage [8]	p = 0.172	p = 0.870	p= 0.434
**Surgery**			
type [9]	p = 0.836	p = 0.896	p = 0.797
EBL [10]	p = 0.452	p = 0.863	p = 0.661
Transfusions (n/y)	p = 0.219	p = 0.139	p = 0.120
Length of surgery [11]	p = 0.613	p = 0.884	p = 0.978
Excised LN [12]	p = 0.138	p = 0.904	p = 0.767
**Adverse clinical outcomes**			
AL (n/y)	p = 0.206	p = 0.765	p = 0.765
RoBF [13]	p = 0.626	p = 0.682	p = 0.067
SSI (n/y)	p = 0.395	p = 0.435	p = 0.204
LoHS [14]	p = 0.488	p = 0.491	p = 0.223

[1] Dichotomized using ≥75 yrs. as a cut-off; [2] dichotomized using ≥25 kg/m^2 as a cut-off; [3] dichotomized using 120 g/L or 130 g/L cut-offs for females and males, respectively; [4] grouped into ASA=1/2 and ASA=3; [5] grouped into G=1/2 and G=3/4; [6] grouped into N0 and N1/2; [7] grouped into tis/T1/T2 and T3/4; [8] grouped into stage 0/I/II and III/IV; [9] open and robotic; [10] dichotomized using ≥150 ml as a cut-off; [11] dichotomized using ≥175 minutes as a cut-off; [12] dichotomized using ≥15 as a cut-off; [13] dichotomized using ≥5 days as a cut-off; [14] dichotomized using ≥6 days as a cut-off. F, females; M, males; ASA, the American Society of Anesthesiologists physical status classification system; G, histological grade; n/y, no/yes; M, distant metastasis; N, lymph node metastasis; T, depth of tumor invasion; EBL, estimated blood loss; LN, lymph nodes; AL, anastomotic leak; RoBF, restoration of bowel function; SSI, surgical site infections; LoHS, length of hospital stay.
